# American Text-Book of Gynecology

**Published:** 1894-01

**Authors:** 


### American Text-Book of Gynecology.

Mr. W. B. Saunders, Publisher, of Philadelphia, Pa., announces this work as ready for early issue. It is the joint work of Drs. Howard Kelley, Pryor, Byford, Baldy, Tuttle, and others who stand before the profession for all that is progressive in gynecology. The work will contain operations not before described in any other book, notably ablation of fibroid uterus. It is designed as a profusely illustrated reference book for the practitioner, and every practical detail of treatment is precisely stated.

